# Percutaneous screw fixation and cementoplasty of metastatic sternal fracture: Descriptions of 2 cases^☆^

**DOI:** 10.1016/j.radcr.2022.03.088

**Published:** 2022-04-20

**Authors:** Quentin Letty, Rémi Grange, Sylvain Bertholon, Fabrice-Guy Barral, Christelle Brosse, Stéphanie Morisson, Nicolas Stacoffe, Sylvain Grange

**Affiliations:** a Department of Interventional Radiology, Saint-Etienne University Hospital, Avenue Albert Raimond, 42055 Saint-Etienne cedex 2, France; b Department of supportive care in Oncology, Saint-Etienne University Hospital, Avenue Albert Raimond, 42055 Saint-Etienne cedex 2, France; c Department of Radiology, Lyon University Hospital, Lyon South Hospital center, 165 Chemin du Grand Revoyet, 69495 Pierre-Bénite, France

**Keywords:** Sternum, Fracture, Internal cemented screw fixation, Interventional Radiology. *Abbreviations:* FICS, Fixation by internal cemented screw, NPRS, Numeric Pain Rating Scale, EQ-5D, EuroQol 5 dimensions score

## Abstract

Secondary lesions and hemopathy localized in sternal bone may be responsible for persistent pain and resistant to classical analgesics. Surgical treatment is not applicable in these cases. We report on 2 cases of sternal osteosynthesis by internal cemented screw fixation, under fluoroscopy and CT scan control, without complication and with clear, immediate reduction of pain. Cementoplasty alone does not appear to be the most appropriate approach for treating lytic sternal lesions which are subject to traction and distraction forces, and resistant to analgesics. Discussion of these 2 cases demonstrate that internal cemented screw fixation allows for rapid management of pain in lytic lesions of the sternum in cancer-related context and should be more widespread in the medical community.

## Introduction

The sternum is a common site for bone metastasis, which most commonly originates from breast, lung, kidney and thyroid cancers, and also from lymphomas and myelomas [1,2]. We report on 2 cases of sternal metastatic fractures treated by imagery guided percutaneous fixation by internal cemented screws (FICS), which others have shown to be efficient for sternum stabilization, and for alleviation of pain symptoms [3].

## Case 1

**Fig. 1:**
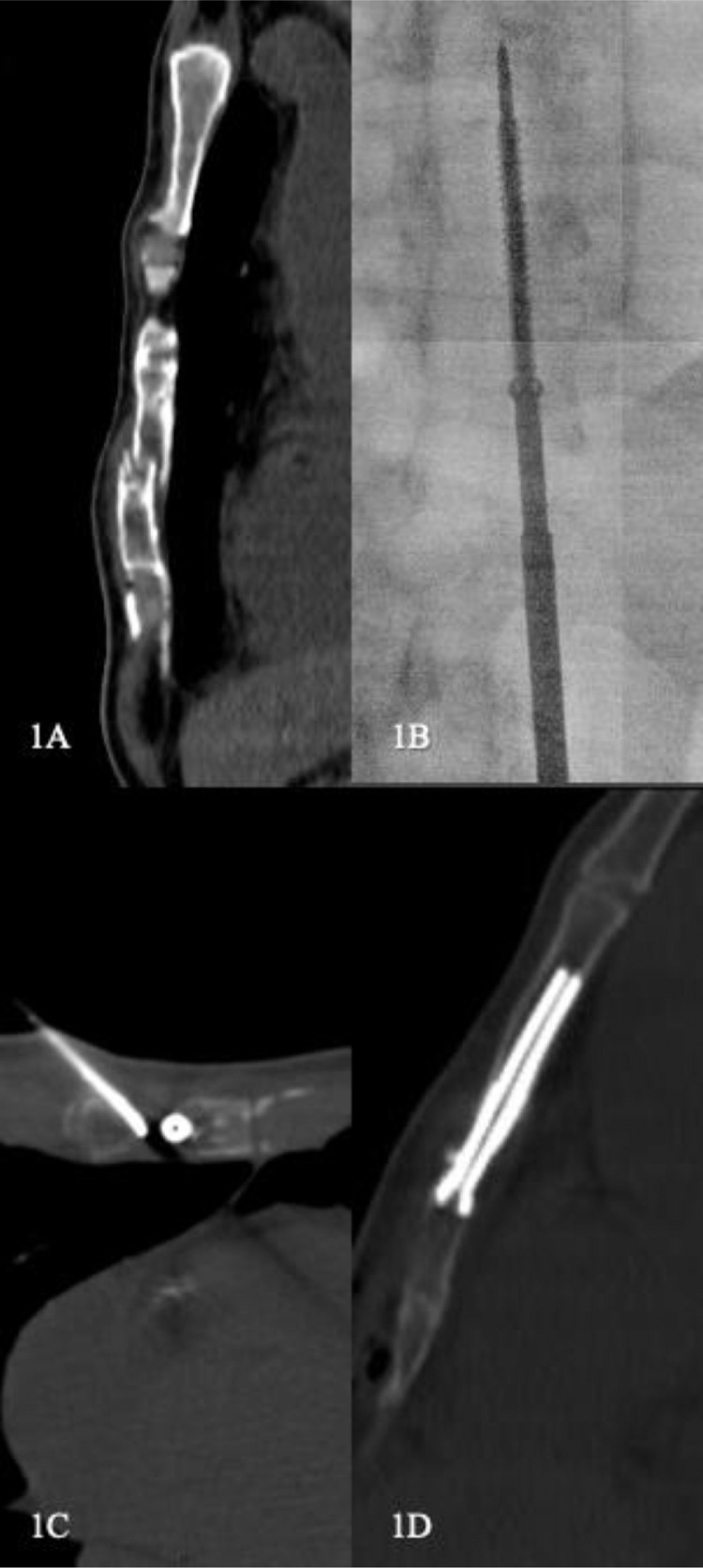
Sixty-six-year-old man with lung carcinoma, sternal metastasis, and a pathologic fracture. (A) Preoperative CT-scan images in sagittal section showing the pathologic fracture with 4 mm cortical displacement. (B) Frontal fluoroscopy showing the screw advance. (C) CT-scan in axial section showing the position of TCD trocar before cement injection (1C). (D) CT-scan sagittal section (1D) showing the result after the procedure, with no leaking of cement, satisfactory fracture stabilization, and no cortical effraction of the screws.

Patient 1, a 66-year-old male patient consulted for thoracic pain following a non–small cell lung carcinoma with multiple osteolytic metastasis located in the spine, right humerus, sternum, and pelvis. The patient reported pain increasing when changing positions, while coughing, and deep breathing. Such symptoms were associated with night pain, causing sleep interruption, and with limitation of the patient's physical activities. The parietal pain led to a CT scan which confirmed the fracture with moderate displacement, with cortical disjunction of 4 mm (Fig. 1A). The patient was taking 30 mg of Oxycodone per os per day to alleviate the chest pain. FICS was decided upon after a multidisciplinary team meeting with oncologists, pain physicians, radiation oncologists, surgeons, and interventional radiologists. The patient was fully informed of the procedure by the interventional radiologist during a medical consultation. The procedure was performed using CT-scan guidance (CONFIDENCE 20, Siemens Healthineers, Princeton, NJ) combined with fluoroscopy (Stephanix, Saint-Etienne, France), under general anesthesia, after intravenous antibiotics (Cefazolin 2 g intravenous), in the dorsal decubitus position. A CT scan without iodinated contrast injection was then performed. Delayed local anesthesia with 10 mL of 2% Naropein was used with a 22G needle on the sternal periosteum. A 1 cm skin incision allowed the placement of the guide wire, navigated longitudinally from entry into the xyphoid appendix to the top of the sternal body with CT scanner, and fluoroscopy guidance. Then, a partially threaded titanium Asnis cannulated screw (Stryker, Kalamazoo, MI), with a diameter of 6.5 mm and a length of 60 mm was inserted through the bone metastasis (Fig. 1B), with a tangential cross of the fracture, its 2 extremities in healthy bone parts. A sternal bone biopsy was performed before installing the screw with an ARROW OnControl 11G 102 mm needle (Teleflex Medical, Athlone, Ireland) using the same method and confirmed the diagnosis of non–small cell lung carcinoma metastasis.

The cementation was performed using 2 14G-125 mm TCD II cementoplasty trocar (Thiebaud, France) by injection of polymethylmethacrylate (PMMA) cement PALACOS R+G with GENTAMICIN (Heraeus Medical GmbH, Wehrheim, Germany) associated with Tungsten, with a transversal approach (Fig. 1C). This allowed us to inject 10 mL of cement to fill in the fractured areas and all the lytic areas, as well as the ends of the screws anchored in the healthy bone. The control CT scan showed no bone displacement with satisfactory screw location and no cortical effraction, nor significant cement leakage (Fig. 1D). The procedure took 2 hours and 5 minutes. The patient was completely pain free immediately after the procedure and was discharged the following day.

The 1-month post-operation pain evaluation on patient 1 using the numeric pain rating scale (NPRS) showed a significantly decrease from 7/10 to 0/10 during sneezing and coughing, from 3/10 to 0/10 for deep breathing, and from 7/10 to 0/10 for nocturnal pain. EuroQol 5 dimensions score (EQ-5D) [4,5] measuring the health-related quality of life increased from 5/100 to 95/100 after the procedure. The same scores were found during a 3-month post-procedure consultation.

## Case 2

**Fig. 2:**
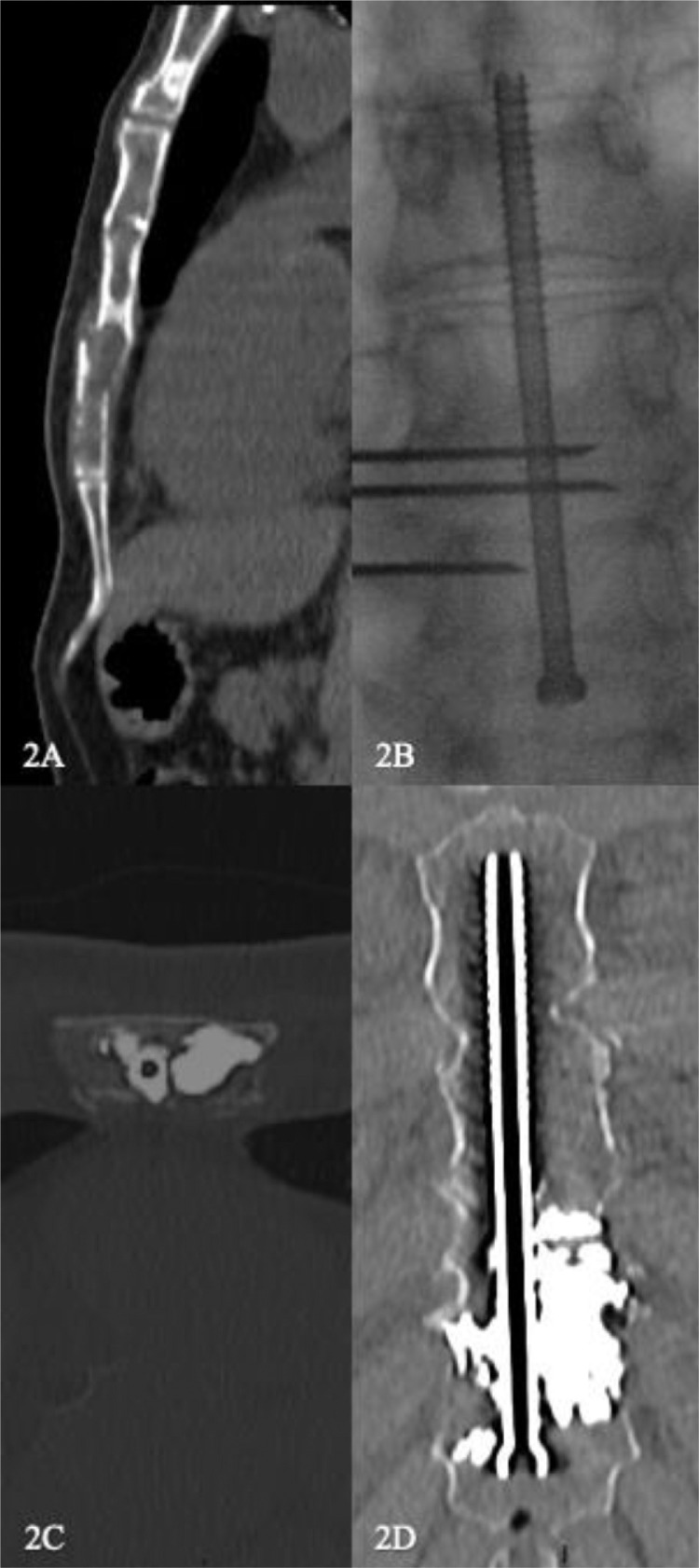
Sixty-eight-year-old man with follicular lymphoma, sternal localization, and a pathologic fracture. (A) Preoperative CT-scan images in sagittal section showing the pathologic fracture with 5 mm cortical disjunction. (B) Frontal fluoroscopy showing location of cementing trocars. (C) CT-scan in axial section showing results after screwing and cementation. (D) CT-scan frontal section showing the result at the end of the procedure, with no leaking of cement, satisfactory fracture stabilization, and no cortical effraction of the screws.

Patient 2, a 68-year-old male with grade 1-2 follicular lymphoma according to WHO classification, had a pathologic fracture of the sternum associated with multiple mediastinal adenomegalies A sternal pathologic fracture was discovered following the patient reporting parietal chest pain and confirmed with CT scanner (Fig. 2A). The scan confirmed the fracture with 5 mm cortical disjunction. The fracture was responsible for the same symptomatology with a significant impact on quality of life (EQ5D scale was 60/100). The patient was using transdermal fentanyl patches at the dose of 12 mg/72 h to alleviate his sternal pain. FICS was decided upon after a multidisciplinary team meeting with oncologists, pain physicians, radiation oncologists, surgeons and interventional radiologists, and after the patient was provided relevant medical information.

The beginning of the procedure was conducted similarly to the process described above for patient 1, under scanographic and scopic control. A partially threaded titanium Asnis cannulated screw (Stryker, Kalamazoo, MI), 95 mm in length and 6.5 mm in diameter, was inserted through the bone metastasis. A single bone biopsy was made and confirmed the diagnosis of follicular lymphoma. Cementation was performed using 3 14G-125 mm TCD II cementoplasty trocar (Thiebaud, France) by injection of polymethylmethacrylate (PMMA) cement PALACOS R+G with GENTAMICIN (Heraeus Medical GmbH, Wehrheim, Germany) associated with Tungsten, with a transversal approach (Fig. 2B). The control CT scan showed no bone displacement and satisfactory screw location with no cortical effraction, nor any significant cement leakage (Figs. 2C and D). The procedure took 2 hours and 10 minutes.

Upon waking up, the patient reported total disappearance of pain, and he left the hospital the day after the procedure. A 1-month CT scan showed no displacement, satisfactory fracture stabilization, and no signs of complications. A 1-month evaluation showed a decrease from 10/10 to 0/10 during sneezing and coughing and a total disappearance of nocturnal pain. Following the procedure, he discontinued painkiller consumption, and resumed physical activity. EQ-5D quality of life scores increased from 60/100 to 80/100. The same scores were found during a 3-month post-procedure consultation.

## Discussion

With aging populations and increased access to new oncologic treatments, bone metastases occur more frequently. They are associated with pain, loss of mobility, and reductions in quality of life. Cementing alone may be effective for alleviating pain but the lack of stabilization appears unsatisfactory for treating long bone fractures which are subjected to torsion and distraction forces and expose the risk of fracture [8,9]. Sternal localization is painful, notably because of the constant movements of the thorax during respiration, coughing, and position changes. Surgery is an invasive treatment option which can be used with curative intentions or for cases of uncontrollable pain [2,6,7].

Management of metastatic sternal fracture by percutaneous image-guided fixation with an internal cemented screw is a rarely used technique because of lack of awareness. However, in the 2 cases we describe, it appears to treat pain effectively for patients. This may provide a rapid recovery time in these vulnerable patients and could result in avoiding delays in chemotherapy treatment. This approach might also lower hospitalization complications by reducing hospitalization time to a minimum and by reducing side effects of high consumption of painkillers [3,10]. The effect on pain is probably due to the reduction of friction and distraction forces by stabilizing the fracture with both screws and cement, combined with cement heat properties causing denervation [3,8]. This treatment seems all the more feasible since only 1 screw seems necessary given the absence of rotational forces on the sternal bone.

The feasibility of such a treatment has been demonstrated in a single series of 9 patients, with high technical and clinical success, and a low rate of complications, with a single hematoma occurring during the biopsy, leading to a 1-day hospitalization extension (grade 2 complication [11]). One patient had another fracture at the osteosynthesis site (grade 4 complication), which was not the case for 2 patients we describe. This previous study showed a significant decrease in pain (5.6 ± 2.8 before and 1.1 ± 1.6 after the procedure, *P* = .03), as assessed by the NPRS score [3].

Two sternal osteosynthesis techniques have been described [3]. The first technique consists of cementing before the screwing. This technique requires rapidity in the execution of the procedure, as the cement solidifies quickly; a maximum time of about 20 minutes is allowed for the procedure. The second technique consists of screwing before cementing. This technique does not require rapid placement of the screw and allows filling of large lytic metastases.

The FICS procedure shows encouraging results and, given the complex forces on the sternum, cemented screws have an advantage over cementing alone in these instances, like the one previously described in the femur [12]. Given the very satisfactory results, the absence of complications, and the lack of satisfactory alternatives described for these 2 patients, more awareness about the potential of the technique of percutaneous screw fixation, and cementoplasty of metastatic sternal fracture is needed among the interventional radiology community.

## Patient consent

Written informed consent was obtained for this case report
